# Cost effectiveness of tac versus fac in adjuvant treatment of node-positive breast cancer

**DOI:** 10.3747/co.v17i1.445

**Published:** 2010-02

**Authors:** N. Mittmann, S. Verma, M. Koo, K. Alloul, M. Trudeau

**Affiliations:** * hope Research Centre, Division of Clinical Pharmacology, Sunnybrook Health Sciences Centre, Toronto, ON; † Department of Medical Oncology, Ottawa Regional Cancer Centre, Ottawa, ON; ‡ Sanofi–Aventis Canada, Laval, QC; § Odette Cancer Centre, Sunnybrook Health Sciences Centre, Toronto, ON

**Keywords:** Adjuvant chemotherapy, breast cancer, cost analysis, economic model, prophylaxis

## Abstract

**Background:**

This economic analysis aimed to determine, from the perspective of a Canadian provincial government payer, the cost-effectiveness of docetaxel (Taxotere: Sanofi–Aventis, Laval, QC) in combination with doxorubicin and cyclophosphamide (tac) compared with 5-fluorouracil, doxorubicin, and cyclophosphamide (fac) following primary surgery for breast cancer in women with operable, axillary lymph node–positive breast cancer.

**Methods:**

A Markov model looking at two time phases—5-year treatment and long-term follow-up—was constructed. Clinical events included clinical response (based on disease-free survival and overall survival) and rates of febrile neutropenia, stomatitis, diarrhea, and infections. Health states were “no recurrence,” “locoregional recurrence,” “distant recurrence,” and “death.” Costs were based on published sources and are presented in 2006 Canadian dollars. Model inputs included chemotherapy drug acquisition costs, chemotherapy administration costs, relapse and follow-up costs, costs for management of adverse events, and costs for granulocyte colony-stimulating factor (g-csf) prophylaxis. A 5% discount rate was applied to costs and outcomes alike. Health utilities were obtained from published sources.

**Results:**

For tac as compared with fac, the incremental cost was $6921 per life-year (ly) gained and $6,848 per quality-adjusted life-year (qaly) gained. The model was robust to changes in input variables (for example, febrile neutropenia rate, utility). When g-csf and antibiotics were given prophylactically before every cycle, the incremental ratios increased to $13,183 and $13,044 respectively.

**Conclusions:**

Compared with fac, tac offered improved response at a higher cost. The cost-effectiveness ratios were low, indicating good economic value in the adjuvant setting of node-positive breast cancer patients.

## 1. INTRODUCTION

The multicentre phase iii randomized Breast Cancer International Research Group (bcirg) 001 trial (“tac–fac”) showed that efficacy with docetaxel (Taxotere: Sanofi–Aventis, Laval, QC) in combination with doxorubicin and cyclophosphamide (tac protocol) was improved over that with the standard protocol of 5-fluorouracil, doxorubicin, cyclophosphamide (fac) for the adjuvant treatment of patients with operable node-positive breast cancer. The study observed 1491 women between the ages of 18 and 70 years with axillary node–positive breast cancer who were randomly assigned to 6 cycles of either tac or fac as adjuvant chemotherapy after surgery. Administration of granulocyte colony–stimulating factor (g-csf) was given to only patients who experienced 1 episode of febrile neutropenia or infection.

As compared with fac, the tac regimen was associated with a higher incidence of febrile neutropenia, but this higher incidence did not result in a significantly different or elevated rate of moderate-to-severe infection in the patients who received tac chemotherapy. During a 5-year follow-up period, the study showed improved disease-free survival (dfs, primary efficacy endpoint) and overall survival (os, secondary endpoint) for patients receiving the tac protocol as compared with those receiving the fac protocol 1. The expansion of the indication for tac from advanced to early breast cancer may lead to an increase in chemotherapy expenditures for hospitals and provincial payers alike. However, recurrences avoided by the use of tac will have an effect in terms of years of life saved and may generate cost savings attributable to the reduction in disease recurrence.

## 2. OBJECTIVE

The objective of the present economic analysis was to compare, from the perspective of Cancer Care Ontario (cco) and the Ontario Ministry of Health and Long-Term Care, the effectiveness, cost, and incremental cost-effectiveness of two adjuvant chemotherapy strategies after primary surgery for breast cancer in women with operable, axillary lymph node–positive breast cancer:

- **tac** Docetaxel 75 mg/m^2^ as a 1-hour intravenous (IV) infusion, in combination with doxorubicin 50 mg/m^2^ as an IV infusion and cyclophosphamide 500 mg/m^2^ as an IV infusion, all given on day 1 every 3 weeks for 6 cycles.
- **fac** 5-Fluorouracil 500 mg/m^2^ as an IV infusion, in combination with doxorubicin 50 mg/m^2^ as an IV infusion and cyclophosphamide 500 mg/m^2^ as an IV infusion, all given on day 1 every 3 weeks for 6 cycles.

## 3. METHODS

The tac–fac study showed that 6 cycles of the tac protocol were superior to 6 cycles of the fac protocol with respect to dfs and os in a group of patients with node-positive breast cancer over a 5-year time horizon 1. The cost of tac is greater than the cost of fac when it comes to acquisition, administration, and adverse event management. Given the superior efficacy but higher cost of tac, a cost-effectiveness analysis was considered a reasonable economic evaluation 2. An incremental cost per life-year (ly) gained was the primary economic outcome. A secondary economic outcome was the incremental cost per quality-adjusted life-year (qaly) gained.

**FIGURE 1:**
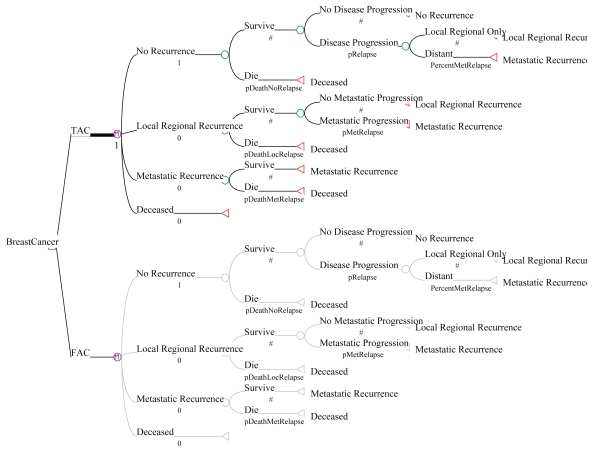
The model as a decision tree. pDeath… = probability of death from the indicated state [No Relapse, Loc (locoregional) Relapse, distant Met (metastatic) Relapse]; pRelapse = probability of relapse from the No Relapse state; pMetRelapse = probability of distant Met relapse from the Loc Relapse state; PercentMetRelapse = proportion of distant Met Relapse.

A Markov model (Figure 1), developed from the perspective of cco and the Ontario Ministry of Health and Long-Term Care, was used to follow a population of patients treated in the tac–fac study [1480 subjects, 99.3% of the overall population; 744 in the tac arm (99.9%), 736 in the fac arm (98.7%); median age: 49.0 years]. Patients in the tac arm experienced 141 recurrences (19.0%) and 162 deaths (21.8%); in the fac arm, the numbers were 195 (26.5%) and 246 (33.4%) respectively 3,4. A cycle length of 6 months was used. The decision–analytic model started at the initiation of adjuvant chemotherapy. The model measured disease recurrences within the remaining years of life for all patients, and it comprises these health states:

- ***No Recurrence:*** No disease progression
- ***Locoregional Recurrence:*** Recurrence without metastases
- ***Distant (Metastatic) Recurrence:*** Recurrence with metastases
- ***Deceased:*** Patient died (from breast cancer cause or other causes)

“Deceased” is an “absorbing state.” Any given woman could stay in the other three “non-absorbing” health states for more than one Markov cycle.

All women start in the No Recurrence state. Any given women could stay at the same state for more than one cycle or progress into the next state, with the final state being Deceased (Figure 1). The lifetime horizon included two time periods, a period of treatment and observation (5 years, corresponding to the length of the tac–fac study) and a period of follow-up for surviving patients (beyond the 5th year until death).

The construct of this model makes certain assumptions. Recurrences (at 5 years) are considered mutually exclusive: patients could have either a Locoregional or Distant (Metastatic) recurrence. Death can occur after the No Recurrence, Locoregional Recurrence, or Distant (Metastatic) Recurrence states. In the base-case analysis, the assumption was made that filgrastim (g-csf) was given secondary to an episode of febrile neutropenia per the tac–fac study protocol, and a standard weight was used for patients. Transfusion rates were based on the percentage of patients that had a “need for blood transfusions” as reported in the tac–fac study 1. The base-case analysis comparing tac with fac was constructed using the entire population of the tac–fac study and g-csf as secondary prophylaxis for febrile neutropenia over a lifetime time horizon.

Two measures of effectiveness (outcome) were considered in the analysis: lys gained (based on study dfs) and qalys gained. Grade 3 or 4 serious adverse events of febrile neutropenia, stomatitis, diarrhea, and infection 1 were included. Results at 5 years from the tac–fac study were used to derive the 6-month constant probabilities of Locoregional Recurrence and Distant (Metastatic) Recurrence, and the 6-month constant probabilities of Death for patients coming from any previous state [No Recurrence, Locoregional Recurrence, and Distant (Metastatic) Recurrence].

Health utilities were not collected during the tac–fac study, and we therefore obtained sources in the literature 5–7. The disutility associated with the use of docetaxel in the tac arm was based on the disutility associated with adverse events in patients who had received docetaxel in the tac–fac study, the probability of an adverse event, the number of adverse events, and the duration of adverse events in that study 1,3. The disutility associated with adverse events was calculated to be 0.0072 for the tac regimen and was found to be 0.0035 8 for the fac regimen, for a difference of 0.0037. In the base case, the utility value for the fac regimen was assumed to 0.72 (reflecting utility values for adjuvant chemotherapy 9) and the utility value for the tac regimen was calculated to 0.7163 (based on the utility value for fac, less the incremental disutilities attributable to the adverse events: 0.72 – 0.0037). Utility values for health states were the same for both groups. For No Recurrence, Locoregional Recurrence, and Distant (Metastatic) Recurrence, utility values were 0.960 5, 0.816 5, and 0.49–0.65 6 (mean: 0.57) 7 respectively.

### 3.1 Costs

Model costs included direct medical costs—namely, drug acquisition costs for the tac and fac treatments; adverse events costs for grades 3 and 4 febrile neutropenia, stomatitis, diarrhea, and infection; and costs of primary and secondary prophylaxis with g-csf. Infusion times for drug administration were considered in the calculation of drug costs. Costs associated with chemotherapy administration, with follow-up, and with Distant (Metastatic) and Locoregional recurrence chemotherapy regimens, supportive care, and diagnostic tests were also considered.

Costs of the assessed treatments included drug acquisition and administration costs, which include 2.5 hours of chair time for tac and 1.5 hours of chair time for fac, with nursing and overhead, for the 6 cycles of treatment. These costs were applied once in the model at the starting point (time 0). Drug acquisition costs were obtained from cco and Sanofi–Aventis Canada.

The model uses 2006 Canadian costs. All non–2006 costs were inflated to 2006 Canadian dollars. Costs and outcomes were discounted at a rate of 5% 2. Nonmedical and indirect costs were not considered in the analysis, per the provincial ministry of health perspective. The cost of capital equipment was not included in this analysis. The equipment to administer tac was assumed to exist in a hospital setting established to administer chemotherapy.

#### 3.1.1 Costs of Chemotherapies for Subsequent Metastatic Disease

After the administration of adjuvant chemotherapy, the costs of subsequent chemotherapy for patients in the metastatic setting were based on advanced breast cancer treatment protocols from the cco Drug Formulary 10. The cost of a treatment comprises drug acquisition costs and chemotherapy administration time. The protocol was multiplied by its percent utilization, and protocols were then stratified into first-line, second-line, and third-line chemotherapy regimens based on cco practice guidelines 10. It was assumed that the costs for fourth- and fifth-line chemotherapy would be the same as for third-line chemotherapy. Table I details the specific regimens given as subsequent lines of therapy in the fac and tac groups. In the tac arm, in the first line, trastuzumab and vinorelbine were given to patients positive for the human epidermal growth factor receptor (her2+), and capecitabine or vinorelbine were given to patients negative for the receptor (her2−). In the fac arm, in the first line, trastuzumab and docetaxel were given to her2+ patients, and docetaxel was given to her2− patients. In the second line, her2+ and her2− patients in both arms were given capecitabine. In the tac arm, her2− patients could receive vinorelbine instead of capecitabine. In the third line, docetaxel was given to her2+ patients in the tac arm, and vinorelbine was given to her2+ patients in the fac arm.

#### 3.1.2 Costs of Supportive Care

In the model, supportive care costs consisted only of concomitant medications used in the administration of chemotherapy (Table I). Costs for transfusions were determined by multiplying the proportion of patients described as having had a “need for a blood transfusion” per the tac–fac study results by the cost of the transfusion. All patients received ondansetron for nausea and dexamethasone; the average cost of ondansetron and dexamethasone per patient was calculated (Trudeau M. Personal communication). The final average supportive care cost per patient entered into the model was $65.08.

#### 3.1.3 Costs of Adverse Events

With respect to adverse events, only grade 3 or 4 events with statistically significant differences between the two evaluated regimens and potentially leading to hospitalization were considered in the model. The adverse events considered were febrile neutropenia, stomatitis, diarrhea, and infection (Table II). All other adverse events reported (for example, anemia, thrombocytopenia, asthenia, vomiting and nausea, abdominal pain, and so on) were assumed to have a marginal effect on the economic outcomes, because they were not statistically different between the two groups 1. The model assumed that the adverse events were considered to be directly associated with the tac and fac chemotherapies and to occur over the treatment period, per the results of the tac–fac study. Codes from the *International Statistical Classification of Diseases and Related Health Problems,* 10th revision, for febrile neutropenia, stomatitis, diarrhea and infection were determined and costed using the Ontario Case Costing Initiative 13. These costs were applied once in the model at the starting point (time 0, corresponding to the advanced treatment phase). The cost of febrile neutropenia included hospitalization costs and treatment of febrile neutropenia with g-csf. Per the tac–fac study, all patients received prophylactic antibiotics 1 at a cost of $314.10. An average adverse event cost per patient was calculated based on the percentage occurrence of each adverse event multiplied by the cost per event. Diagnostic and procedural costs associated with the treatment of breast cancer are listed in Table I.

#### 3.1.4 Follow-Up Costs

Information on follow-up procedures and the frequency of those procedures was validated by the clinical authors of this article. Associated costs were obtained from the Ontario Ministry of Health and Long-Term Care *Schedule of Benefits* 16. Procedures included physician or oncologist visits, mammograms, laboratory testing, and radiology. Annual costs were determined by multiplying costs per episode by the annual frequency of the procedure, and a total annual cost of all follow-up procedures was obtained.

For women with Distant (Metastatic) Recurrence, the total annual cost of follow-up was determined to be $1,729.10.

Follow-up costs for women without recurrence of breast cancer included physician assessments, clinical costs (including overhead), hematology, biochemistry, bone scan, chest radiographs, liver ultrasound, and annual mammogram 15. The cost was stated as $582.82 in year 1, declining to $356.93 by year 5, after inflation. It was assumed that years 1–3 had a follow-up cost of $582.82 and that all years thereafter had a cost of $356.93.

Follow-up costs for women with Locoregional Recurrence were $1,032.76 for years 1 and 2, $764.27 for years 3 and 4, and $620.65 for years 5 and beyond, after inflation. The annual cost was $805.89, based on an average of 6 years (Table I).

#### 3.1.5 Cost of Secondary Prophylaxis with G-CSF after a First Episode of Febrile Neutropenia

The average cost per patient for secondary prophylaxis with g-csf was determined by multiplying the cost per event by the proportion of patients who received any secondary prophylaxis by the average number of cycles for which they received secondary prophylaxis (Table III). Detailed secondary prophylaxis information was not available from the publication. Here, the unpublished clinical study report was used to provide the data. The average cost per patient of secondary prophylaxis was considered only once for each arm of treatment at the starting point of the model (time 0, corresponding to the adjuvant treatment phase).

#### 3.1.6 Cost of Primary Prophylaxis of Febrile Neutropenia with G-CSF

In the tac–fac study, patients who experienced febrile neutropenia received g-csf (secondary prophylaxis). We wanted to conduct an alternative analysis looking at the cost-effectiveness of tac versus fac in the context of primary g-csf prophylaxis—that is, the administration of g-csf before the occurrence of febrile neutropenia or other documented infection. To do so, we used the geicam (Grupo Español de Investigación del Cáncer de Mama) 9805 study 17 for the costing of adverse events, because we could then compare adverse events rates with and without primary prophylaxis within the context of a single study. Because the rate of febrile neutropenia in the tac group was the major driver of adverse event costs and the variable that would be affected by g-csf prophylaxis, it is important to note that the adverse event rates for grades 3 and 4 febrile neutropenia reported in the geicam study for the pre-tac and fac groups were similar to those reported in the tac–fac study 1. In this scenario, as in our base-case analysis, all patients received antibiotic prophylaxis.

### 3.2 Sensitivity Analysis

Sensitivity analyses (one-way and bootstrapping) were conducted to test the robustness of results with regard to variations in key parameters considered in the model. Sensitivity analyses were conducted for adverse event rates, the rate of febrile neutropenia, the proportion of recurrences in the Locoregional and Distant (Metastatic) recurrence states, follow-up and supportive care costs, utility values, and transition probabilities. Table IV outlines the specific analyses.

#### 3.2.1 Analysis

An incremental cost-effectiveness analysis was conducted for the base-case analysis. A number of one-way and bootstrapping sensitivity analyses were conducted as described in the preceding subsection. Additionally, an alternative analysis using g-csf as primary prophylaxis was conducted, and sensitivity analyses were built around that model as well.

A number of assumptions were made in constructing this model:

- Transition probabilities were constant over time. Because recurrence rates were available only at 5 years, 5-year rates were converted into 6-month probabilities such as the probability of any single patient having a recurrence in any given model cycle (semester). The formula for the time conversion is
  $$1-{\text{e}}^{-\text{rate}*\text{time}}.$$
- For instance, if 30% of women experienced a recurrence at 5 years (ten Markov cycles), the constant 6-month transition probability was
  $$0.029\;[1-{\text{e}}^{-0.3*1/10}]$$
- Recurrences were considered mutually exclusive (at 5 years). Patients could either have a Locoregional Recurrence or a Distant (Metastatic) Recurrence.
- Death could occur after the No Recurrence, the Locoregional Recurrence, or the Distant (Metastatic) Recurrence states
- In the base-case analysis, we assumed that the costs of fourth- and fifth-line chemotherapy regimens were identical to those of third-line chemotherapy.
- In the base-case analysis, we assumed that filgrastim (g-csf) was given secondary to an episode of febrile neutropenia, per the tac–fac study.
- A weight of 60 kg and 7 days of g-csf therapy per cycle were assumed for patients receiving g-csf.
- Only grade 3 or 4 adverse events with statistically significant differences were considered in the analysis. These included febrile neutropenia, infection, stomatitis, and diarrhea (Table V).
- For patients experiencing anemia, only needed blood transfusions were costed and included in the analysis.

At baseline, the median age of patients in the tac–fac study was 49 years. The duration of the study was 5 years. Life expectancy was extrapolated based on the Wisconsin population for a woman starting at 55 years of age 18, corresponding approximately to the mean age of the women completing the clinical trial. A life expectancy was assigned to each patient according to health status at the end of the tac–fac clinical trial time horizon. It should also be noted that the adverse event rates used in the primary g-csf prophylaxis sensitivity analysis were taken from the geicam study, because those rates provided information about adverse events after primary prophylaxis (Table V).

## 4. RESULTS

The base-case analysis used in this model applied a 5% discount rate, the Canadian costs for chemotherapies recommended by cco guidelines 10, and febrile neutropenia rates based on the available tac–fac study results 17. The incremental cost-effectiveness ratio (icer) for tac compared with fac was $6,921.24/ly gained. The incremental cost–utility ratio (icur) for tac compared with fac was $6,848.39/qaly (Table IV).

Several one-way and probabilistic sensitivity analyses were conducted. Adverse event rates used in the base-case model were altered in a variety of ways in the sensitivity analyses. When the rates were increased by 25%, the incremental ratios were $7,129.14/ly gained and $7,054.09/qaly gained. When decreased by 25%, the incremental ratios were $6,713.35/ly gained and $6,642.68/qaly gained. When adverse event rates were increased and decreased by 25% in the tac group only, the icers were $7,188.70/ly gained and $6,653.78/ly gained for tac and fac respectively, and the icurs were $7,113.03/qaly and $6,583.74/qaly respectively. Febrile neutropenia rates were increased and decreased by 25%, giving incremental ratios of $7,076.75/ly and $7,002.26/qaly gained and $6,765.73/ly and $6,694.52/qaly gained for tac and fac respectively.

The bootstrapping sensitivity analysis examined a lower and an upper bound for the cost per dfs. The icers ranged from $3,132.16/dfs for the best-case scenario to $20,370.59/dfs for the worst-case scenario. The icurs ranged from $3,060.59/qaly for the best-case scenario to $20,036.64/qaly for the worst-case scenario.

When the model was run using the adverse events observed in the geicam study, in which no primary g-csf prophylaxis was allowed, the icer was $6,893.22/ly gained and the icur was $6,820.66/qaly gained for tac compared with fac. The results of this analysis were very comparable to those based on the tac–fac study, which supports our rationale for using the adverse events rates in the geicam study to evaluate the effect of primary g-csf prophylaxis on the cost-effectiveness of the tac regimen. When g-csf was given as primary prophylaxis, the incremental costs were $13,183.26/ly and $13,044.49/qaly gained for tac compared with fac. The overall cost and the incremental benefit were both higher as compared with the base-case results when g-csf was given prophylactically before each cycle.

Table IV also outlines additional sensitivity analyses (including relapse rate, follow-up costs, ratio of recurrence, and utility) and additional analyses with g-csf as a primary prophylaxis. Increases in the costs associated with follow-up of patients with No Recurrence are observed to result in increased icers, whereas increases in the costs associated with follow-up of patients with Distance (Metastatic) Recurrence are observed to result in a decreased icer, but to a far lesser extent than the increase seen with No Recurrence.

## 5. DISCUSSION

Based on the literature, tac offers improved dfs compared with fac, at a higher cost 3. Patients receiving tac had a 6-month greater life expectancy than did patients receiving fac. The incremental cost-effectiveness ratios for tac compared with fac ($6,921.24/ly gained and $6,848.39/qaly gained) indicate good economic value for tac treatment in the adjuvant setting of node-positive breast cancer patients. Life expectancies adjusted by utilities were lower than the unadjusted life expectancies, but the difference in qalys gained between the two study groups was greater than the observed difference in lys gained, explaining why the cost per qaly gained is lower than the cost per ly gained. According to this model, and based on its assumptions, tac is a cost-effective treatment for adjuvant treatment of breast cancer. Results show that adjuvant tac—with secondary and primary prophylaxis—provides good economic value for women with node-positive breast cancer.

The major cost drivers of this model are the drug acquisition costs of tac and the proportion of patients who achieved dfs. In general, the sensitivity analyses indicated that the results were robust to change. Variations in chemotherapy costs, follow-up costs, supportive care costs, and utility values resulted in relatively similar incremental ratios for the cost per ly gained and the cost per qaly gained. Variations in rates of dfs had a significant effect on the incremental ratios—namely, poor dfs would result in a higher incremental ratio.

Our results were consistent with those from Au *et al.* 21, who reported a cost per qaly of $18,505.54. They used Alberta provincial costs and also based their analyses on the tac–fac study. A similar study in the United Kingdom, with the same clinical trial data, also found that tac was more cost effective than fac. A model was also constructed around the data from the bcirg 001 trial to estimate the cost-effectiveness of tac compared with fac as adjuvant therapy for node-positive breast cancer. Parameters and sensitivity analyses were built around adverse events, cost of chemotherapy and support, survival estimates, utility weights, and costs of monitoring and care after relapse. The cost-effectiveness of tac compared with fac was £15,418/ly gained and £18,188/qaly gained. That study also looked at g-csf as primary prophylaxis and found an increase to £29,432/qaly gained. Although the icer was reported to be higher, the overall conclusions were similar to our own. The higher values may have resulted from the inclusion of additional adverse events such as anemia, pain, and vomiting. And because the analysis reflected the perspective of the U.K. National Health Service, differences in the cost of follow-up and community care may also have affected the icer 22.

Our study has a number of limitations. As used in the model, the clinical data from the tac–fac trial and the assumptions used when trial data were not available to estimate long-term costs may not be representative of real-life experience. Hormonal therapies were not included in the calculation of the cost-effectiveness because, per the tac-fac study, tamoxifen was administered on completion of chemotherapy to patients with estrogen or progesterone receptor–positive (or both) tumours. This treatment strategy would have been applied to both groups, and thus would have not affected the results for one therapy or the other.

The relative cost-effectiveness of fec-d (5-fluorouracil, epirubicin, cyclophosphamide, followed by docetaxel), a more commonly used regimen relative to tac, can be inferred from the results presented here and the 2008 cost-effectiveness analysis by Younis *et al.* 23, who compared the cost-effectiveness of fec-100 (5-fluorouracil, epirubicin, cyclophosphamide) with that of fec-d in women with node-positive breast cancer on adjuvant chemotherapy after surgical treatment. Their study reported an incremental cost difference of $3,544 per patient and an icur of $14,612/qaly gained 23. They used a Markov model and reported the incremental cost utility over a 10-year horizon. Considering that the tac regimen has shown efficacy comparable to that of the fec-100 regimen, per the 5-year dfss and oss observed in the bcirg 001 and pacs 01 studies, and that the tac regimen is, in general, more costly than the fec-100 regimen, the fec-d regimen could be expected to show a cost-effectiveness ratio comparable to the one reported by Younis *et al.* or even better when compared with the tac regimen.

## 6. CONCLUSIONS

Clinical results have shown that tac is superior to fac in terms of the primary efficacy endpoint of dfs and the secondary endpoint of os. The tac regimen offered improved response at a higher cost than that for fac. The incremental cost was $6,921.24/ly gained and $6,848.39/qaly gained for tac as compared with fac when secondary g-csf prophylaxis is given. These costs increase to $13,183.26/ly gained and $13,044.49/qaly gained when primary g-csf prophylaxis is given in the tac group. Overall, the cost-effectiveness ratios for tac compared with fac are low, indicating good economic value for the tac treatment in the adjuvant setting of node-positive breast cancer patients.

## Figures and Tables

**TABLE I:** Cost of drug treatment, chemotherapy regimens, supportive care, adverse events, diagnostic procedures, and follow-up procedures, Canadian dollars

Variable	tac	fac		Source
*Cost of drug treatment*				
Drug acquisition (6 cycles)	9,024.00	301.92		Cancer Care Ontario, 2006 9
Chemotherapy administration (6 cycles)	1,522.08	913.26		Chair time: Cancer Care Ontario Drug Formulary Nursing and overhead costs: 2002 costs^a^ ($35/h and $57.42/h respectively) inflated to 2006 using the Bank of Canada inflation calculator 11
TOTAL	10,546.08	1,215.18		Drug acquisition plus chemotherapy administration
*Chemotherapy regimens*				
First-line	10,686–54,264^b^	11,700–47,892^c^	}	Cancer Care Ontario, 2006 9 (range for her2− to her2+)
Second-line	3,300–10,686^d^	3,300^e^		
Third-line	18,072^f^	11,700^g^		Cancer Care Ontario, 2006 9 (her2+)
*Supportive care cost components*				
Transfusion	41.60	13.56	}	
Ondansetron	31.50	31.50		Sunnybrook Health Sciences Centre, 2005 12
Dexamethasone	6.00	6.00		
TOTAL	79.10	51.06		Derived from the foregoing category costs and the proportion of patients receiving the therapies
Average supportive care cost used in the model	65.08	65.08		Average supportive care cost for the tac and fac arms
*Cost of adverse events*				
Febrile neutropenia	2,367.23	2,367.23	}	
Stomatitis	3,151.18	3,151.18		Ontario Case Cost Initiative, 2005 13
Diarrheas	2,760.30	2,760.30		Bank of Canada inflation calculator 11
Infections	2,367.30	2,367.30		
g-csf acquisition cost for secondary prevention of febrile neutropenia	1,239.77	1,239.77		Sunnybrook Health Sciences Centre, 2005 12 Lalami *et al.,* 2004 14
*Cost of diagnostic procedures performed at diagnosis*				
TOTAL	643.04	643.04		Procedures from Will et al., 2000 15 Ontario Ministry of Health and Long-Term Care, 2004 16 (validated by the clinical authors of this manuscript)
*Total cost of laboratory tests*				
Total cost of all blood work	41.95	41.95		Ontario Ministry of Health and Long-Term Care, 2004 16
*Follow-up costs*^h^				
Total average annual follow-up cost for years 1–3 for patients without breast cancer recurrence	582.82	582.82		Will *et al.,* 2000 15 ($467 non-inflated) Bank of Canada inflation calculator 11 (inflated to $582.82)
Total average annual follow-up cost for years beyond year 3 for patients without breast cancer recurrence	356.93	356.93		Will *et al.,* 2000 15 ($286 non-inflated) Bank of Canada inflation calculator 11 (inflated to $356.93)
Total average annual follow-up cost for patients with distant (metastatic) breast cancer	1,729.10	1,729.10		Costs and codes from Ontario Ministry of Health and Long-Term Care, 2004 16 (procedure validated by the clinical authors of this manuscript)
Total average annual follow-up cost for patients with locoregional breast cancer recurrence	805.89	805.89		Will *et al.,* 2000 15 ($827 for years 1 and 2, $612 for years 3 and 4, $497 for years 5 and 6) Bank of Canada inflation calculator 11 (inflated to $1,032.76 for years 1 and 2, to $764.27 for years 3 and 4, to $620.65 for years 5 and 6) Annual cost based on an average of 6 years

a Risebrough NA, Imrie K, Seung SJ, *et al.* An observational study of resource use and outcomes in indolent follicular non-Hodgkin’s lymphoma for Canada. Unpublished data. 2002.

b her2−: capecitabine or vinorelbine; her2+: trastuzumab plus vinorelbine.

c her2−: docetaxel; her2+: trastuzumab plus docetaxel.

d her2−: capecitabine or vinorelbine; her2+: capecitabine.

e her2+: capecitabine.

f her2+: docetaxel.

g her2+: vinorelbine.

h Follow-up care includes physician assessments, clinic costs (including overhead), hematology, biochemistry, bone scan, chest radiography, liver ultrasonography, and annual mammogram 15.

tac = docetaxel, doxorubicin, cyclophosphamide; fac = 5-fluorouracil, doxorubicin, cyclophosphamide; her2− = disease negative for the human epidermal growth factor receptor; her2+ = disease positive for the human epidermal growth factor receptor.

**TABLE II:** Percentage of grades 3 and 4 adverse events 1

Regimen	Treated population (*n*)	Adverse events (%)			
		Febrile neutropenia^a^	Mucositis and stomatitis^a^	Diarrhea^a^	Infections^a^,^b^
tac	744	24.7	7.10	3.80	3.90
fac	736	2.50	2.00	1.80	2.20

a Extracted from Martin *et al.,* 2005, Table 3, p. 2310 1.

b Non-hematologic infections.

tac = docetaxel, doxorubicin, cyclophosphamide; fac = 5-fluorouracil, doxorubicin, cyclophosphamide.

**TABLE III:** Secondary granulocyte colony–stimulating factor (g-csf) prophylaxis^a^

	tac	fac
Treated population (*n*)	744	736
Patients receiving prophylaxis [*n* (%)]	217 (29.17)	41 (5.57)
Cycles with prophylaxis [*n* (%)]	799 (18.7)	126 (2.9)
Average cycles under prophylaxis (*N*/*n*)	3.7 (799/217)	3.1 (126/41)
Average prophylaxis administrations per patient (*N*/*n*)	1.07 (799/744)	0.17 (126/736)

a From Aventis Pharma Research and Development, 2004, Table 78, p. 193 3.

tac = docetaxel, doxorubicin, cyclophosphamide; fac = 5-fluorouracil, doxorubicin, cyclophosphamide.

**TABLE IV:** Incremental cost-effectiveness ratio (icer) and incremental cost–utility ratio (icur) summary table, Canadian dollars

Variable and variations	icer (CA$)	icur (CA$)
Base case	6,921.24	6,848.39
Adverse event rates (geicam study 19)	6,893.22	6,820.66
Adverse event rates		
Rates increased by 25%	7,129.14	7,054.09
Rates decreased by 25%	6,713.35	6,642.68
Rates in tac increased by 25%	7,188.70	7,113.03
Rates in tac decreased by 25%	6,653.78	6,583.74
Rates of febrile neutropenia increased by 25%	7,076.75	7,002.26
Rates of febrile neutropenia in decreased by 25%	6,765.74	6,694.52
Relapse rate		
Increased probability of relapse by 25% in tac arm	21,126.10	21,126.10
Decreased probability of relapse by 25% in tac arm	2,715.32	2,661.37
Follow-up cost		
No recurrence + 25% metastatic − 25%	7,148.54	7,073.29
No recurrence − 25% metastatic + 25%	6,693.94	6,623.48
Ratio of recurrence		
Recurrence at 25%	7,203.09	7,127.26
Recurrence at 100%	6,357.56	6,290.63
Utility		
Equate utility value	6,921.24	6,777.05
Lowest utility value	6,921.24	7,393.15
Highest utility value	6,921.24	6,571.69
Bootstrapping		
Best case	3,132.16	3,060.59
Worst case	20,370.59	20,036.64
Primary prophylaxis (g-csf)	13,183.26	13,044.49
Adverse event rates (geicam study rates 19)		
Rates increased by 25%	13,277.28	13,137.52
Rates decreased by 25%	13,089.32	12,951.54
Rates in tac increased by 25%	13,355.05	13,214.47
Rates in tac decreased by 25%	13,011.47	12,874.51
Rates of febrile neutropenia increased by 25%	13,265.49	13,125.86
Rates of febrile neutropenia decreased by 25%	13,101.03	12,963.12
Relapse rate		
Increased probability of relapse by 25% in tac arm	37,736.72	37,736.72
Decreased probability of relapse by 25% in tac arm	6,581.31	6,411.48

tac = docetaxel, doxorubicin, cyclophosphamide; g-csf = granulocyte colony–stimulating factor.

**TABLE V:** Percentage of grades 3 and 4 adverse events^a^

Adverse events	tac^b^ [% (*n* evaluable pts)]		fac^c^
	Pre	Post	[% (*n* evaluable pts)]
Febrile neutropenia (per protocol, in 1 or more cycles)^d^	24.6 (114)	6.50 (519)	2.30 (519)
Mucositis and stomatitis 20,e	6.40 (109)	2.60 (111)	2.70 (111)
Diarrhea 19,f	7.00 (114)	2.60 (519)	0.80 (519)
Infections 19,f	2.80 (109)	1.70 (111)	1.80 (111)

a From Martin *et al.,* 2006, Table 3 17.

b Rates without (Pre) and with (Post) primary granulocyte colony–stimulating factor prophylaxis.

c Rates with granulocyte colony–stimulating factor given as secondary prophylaxis.

d Protocol definition implies a fever of 38.1°C or higher, with grade 4 neutropenia requiring intravenous antibiotics or hospitalization (or both), in the same cycle.

e Based on 448 randomized subjects in total (p. 65, Table 14 17).

f Grades 3 and 4 toxicity, with more final results. Only grades 2–4 diarrhea were reported earlier.

tac = docetaxel, doxorubicin, cyclophosphamide; pts = patients; fac = 5-fluorouracil, doxorubicin, cyclophosphamide.
